# Can Cross-Country Genomic Predictions Be a Reasonable Strategy to Support Germplasm Exchange? – A Case Study With Hydrogen Cyanide in Cassava

**DOI:** 10.3389/fpls.2021.742638

**Published:** 2021-12-08

**Authors:** Lívia Gomes Torres, Eder Jorge de Oliveira, Alex C. Ogbonna, Guillaume J. Bauchet, Lukas A. Mueller, Camila Ferreira Azevedo, Fabyano Fonseca e Silva, Guilherme Ferreira Simiqueli, Marcos Deon Vilela de Resende

**Affiliations:** ^1^Department of Plant Science, Universidade Federal de Viçosa, Viçosa, Brazil; ^2^Embrapa Mandioca e Fruticultura, Cruz das Almas, Brazil; ^3^Department of Plant Breeding and Genetics, Cornell University, Ithaca, NY, United States; ^4^Boyce Thompson Institute, Ithaca, NY, United States; ^5^Department of Statistics, Universidade Federal de Viçosa, Viçosa, Brazil; ^6^Department of Animal Science, Universidade Federal de Viçosa, Viçosa, Brazil; ^7^Department of Forestry Engineering, Universidade Federal de Viçosa, Viçosa, Brazil; ^8^Embrapa Café, Universidade Federal de Viçosa, Viçosa, Brazil

**Keywords:** *Manihot esculenta*, cross predictions, population structure, haplotype prediction, breeding, cyanide content

## Abstract

Genomic prediction (GP) offers great opportunities for accelerated genetic gains by optimizing the breeding pipeline. One of the key factors to be considered is how the training populations (TP) are composed in terms of genetic improvement, kinship/origin, and their impacts on GP. Hydrogen cyanide content (HCN) is a determinant trait to guide cassava’s products usage and processing. This work aimed to achieve the following objectives: (i) evaluate the feasibility of using cross-country (CC) GP between germplasm’s of Embrapa Mandioca e Fruticultura (Embrapa, Brazil) and The International Institute of Tropical Agriculture (IITA, Nigeria) for HCN; (ii) provide an assessment of population structure for the joint dataset; (iii) estimate the genetic parameters based on single nucleotide polymorphisms (SNPs) and a haplotype-approach. Datasets of HCN from Embrapa and IITA breeding programs were analyzed, separately and jointly, with 1,230, 590, and 1,820 clones, respectively. After quality control, ∼14K SNPs were used for GP. The genomic estimated breeding values (GEBVs) were predicted based on SNP effects from analyses with TP composed of the following: (i) Embrapa genotypic and phenotypic data, (ii) IITA genotypic and phenotypic data, and (iii) the joint datasets. Comparisons on GEBVs’ estimation were made considering the hypothetical situation of not having the phenotypic characterization for a set of clones for a certain research institute/country and might need to use the markers’ effects that were trained with data from other research institutes/country’s germplasm to estimate their clones’ GEBV. Fixation index (F_ST_) among the genetic groups identified within the joint dataset ranged from 0.002 to 0.091. The joint dataset provided an improved accuracy (0.8–0.85) compared to the prediction accuracy of either germplasm’s sources individually (0.51–0.67). CC GP proved to have potential use under the present study’s scenario, the correlation between GEBVs predicted with TP from Embrapa and IITA was 0.55 for Embrapa’s germplasm, whereas for IITA’s it was 0.1. This seems to be among the first attempts to evaluate the CC GP in plants. As such, a lot of useful new information was provided on the subject, which can guide new research on this very important and emerging field.

## Introduction

Cassava (*Manihot esculenta* Crantz) is an important staple root crop, a food source for millions of people mostly in developing countries, and used for several industrial applications. Subsistence farmers rely on cassava as a vital source of dietary energy, as it can be planted and harvested throughout the year, tolerates periods of drought, and grows on marginal soils (Hillocks et al., 2002). It has been suggested that cassava can be highly resilient in a climate change scenario; providing adaptation opportunities that are not offered by any other basic food crops (Jarvis et al., 2012).

Despite the exciting crop’s future prospect, cassava roots, peels and leaves contain two cyanogenic glucosides, linamarin, and lotaustralin, mainly linamarin. Linamarin and lotaustralin, in the acids and enzymes presence, go under hydrolysis that leads to CN^–^ production and, consequently, hydrogen cyanide (HCN) (Cereda and Mattos, 1996). Although cyanogenic glycosides play important roles such as defense against herbivores and transport forms of reduced nitrogen in some plant species (Poulton, 1990; White et al., 1998), the high content of cyanogenic glycosides (consequently liberated HCN), together with the rapid tuber perishability, represents one of the main shortcomings associated with cassava consumption.

Hydrogen cyanide is a toxic compound that may be harmful to human and animal health, the potential toxicity depends on the amount, route, and duration of the exposure. The toxicological effects of cyanide absorption due to intake of improperly processed cassava occur in acute and chronic form, it can affect the nervous, respiratory, cardiovascular, and endocrine systems (Simeonova et al., 2004), causing symptoms like headache, dizziness, nausea, vomiting, weakness, and among others.

Cassava varieties are classified as bitter and sweet depending on the content of HCN in the roots. This component varies substantially with plant variety, environment conditions, time of harvest, and post-harvest practices (Ojo et al., 2013). Those with concentrations of cyanogenic glycosides greater than 50 mg/kg HCN on a fresh weight basis are classified as bitter cassava, whereas sweet varieties contain less than 50 mg/kg (Obi et al., 2019). Bitter varieties require more effort in terms of processing. Processing is effective to ensure reduction of cyanogenic compound content in cassava food products, as examples of processing steps: peeling, grating, soaking, boiling, cooking, and among others, the combination of procedures within the production system essentially varies depending on the intended end-product, according to the Code of Practice for the Reduction of Hydrocyanic Acid (HCN) in Cassava and Cassava Products (Philippine National Standard, 2020). Besides the most obvious relevance of the identification of sweet cassava varieties, which is to ensure safe recommendations for food consumption, Obueh and Kolawole (2016) described in a comparative study that sweet varieties are also associated with high nutritional quality (Ca, Mg, P, Zn, Cu, and amino acid contents) and low anti-nutrients content (oxalate, tannin, phytate, alkaloid, lignin, and saponin).

Traditional cassava genetic improvement strategies are still very demanding in terms of financial resources; and, historically, cassava is a crop that receives less investment than other commodity crops (Bart and Taylor, 2017). However, this scenario is changing, possibly encouraged by the following reasons: (i) progress constraints of conventional breeding due to the crop biology – e.g., long breeding cycle, slow multiplication rate (Ceballos et al., 2020), (ii) the importance of cassava for food security and poverty alleviation, (iii) the increase of challenges in the face of biotic stresses and, also, (iv) the increased commercial interest in cassava, due to the better starch properties than cereals. Promising results with a recently sequenced genome (Prochnik et al., 2012; Bredeson et al., 2016) and SNP-based genetic linkage maps (International Cassava Genetic Map Consortium [ICGMC], 2014), was obtained in a short period of time, opening a path to the study of key traits’ genetic architecture through genome-wide association studies (GWAS) (Esuma et al., 2016; Wolfe et al., 2016; Brito et al., 2017; Rabbi et al., 2017; Kayondo et al., 2018; Somo et al., 2020; Ogbonna et al., 2021b) and trait improvement by means of genomic predictions (GP) and selection (Oliveira et al., 2012; Okeke et al., 2017; Wolfe et al., 2017; Elias et al., 2018; Andrade et al., 2019; Ikeogu et al., 2019; Torres et al., 2019; Somo et al., 2020; Yonis et al., 2020).

Among the methodologies currently used to accelerate genetic gain and to shorten the breeding cycle interval, genomic selection proposed by Meuwissen et al. (2001) offers great opportunities. Besides the reduction in the selection interval, Werner et al. (2020) pointed out two other additional key advantages: the increased selection accuracy and the possibility of increasing the selection intensity. Genomic prediction can also be extremely useful when applied for a trait that is expensive and/or hard to measure, restricting the phenotypic data’s availability to a subset of the population (De Haas et al., 2012).

Implementation of genomic prediction/selection schemes was recently boosted by the advent of low-cost SNPs marker platforms such as GBS (Elshire et al., 2011). Genotyping must be sufficiently dense to get SNPs that are in linkage disequilibrium with most of the loci controlling important quantitative traits (Okeke et al., 2017). Despite being a trivial practice for some plant breeding programs, and especially for animal breeding programs, some plant breeding programs are still facing the transition’s challenges from conventional plant breeding to a present scenario where conventional breeding is still essential but now it can also be supported by genome-assisted breeding pieces of evidence to drive decision making, by adding relevant information to speed up the breeding pipeline.

Selection decisions are based on genomic breeding values (GEBVs), which are calculated by first estimating the SNP effects in a training population (reference) with both phenotypic and genotypic information available; and then, the SNP effects are multiplied by the genotypes’ marker matrix of a testing population (target) and summed to form the GEBVs (Pryce et al., 2011). One of the essential factors to be considered is how the training sets are composed; they can be constituted of landraces and/or modern improved lines, clones from different breeding programs, across breeding stages, and/or even from different countries (Somo et al., 2020).

Joint evaluations can be desirable under certain situations, its practice is currently more common in animal breeding as crossbred predictions (Pocrnic et al., 2019; VanRaden et al., 2020). However, for plants, recently Somo et al. (2020) attempted to examine diverse cassava germplasm assembled from two breeding programs in Tanzania at different breeding stages to predict traits. In their work, the use of clones in the same breeding stage to build TPs provided higher prediction accuracy than TPs with clones from multiple breeding stages together. They also included a Ugandan TP in either Tanzanian population which did not improve prediction accuracies.

This work aimed to achieve the following objectives: (i) evaluate the feasibility of cross-country genomic predictions between germplasm’s accessions from Embrapa (Cruz das Almas, Brazil) and IITA (Ibadan, Nigeria) for HCN (taking into consideration the hypothetical scenario of lack of phenotypic characterization of clones from a certain research institute/country and the need of predicting their GEBVs with SNPs effects predicted with a TP consisted of clones from other research institutes/countries, which may contemplate cases of interest, e.g., plant diseases); (ii) identify the HCN variability in the cassava germplasm; (iii) provide an assessment of population structure in the joint dataset (Embrapa+IITA); (iv) estimate the genetic parameters of GP based on a haplotype-approach and compare them to those based on single markers. This seems to be among the first attempts to evaluate the cross-country genomic selection in cassava.

## Materials and Methods

### Plant Material

A total of 1,230 cassava clones from the Cassava Breeding Program (CBP) of Embrapa Mandioca e Fruticultura (Cruz das Almas, Brazil) were genotyped and phenotyped for HCN. The set of clones consisted of landraces and modern breeding lines, collected from different Brazilian growing regions. It consisted of a selected subset from a panel of 1,536 clones, which were revealed as a unique subset out of an original set of 3,354 clones by identity-by-state analysis carried out by Ogbonna et al. (2021a). The dataset from the International Institute of Tropical Agriculture (IITA) was obtained from the open-source cassava breed base instance^1^, it included the genotypic and phenotypic information of 590 cassava clones evaluated in Ibadan, Nigeria.

### Phenotypic Characterization of Hydrogen Cyanide Content

Field trials were carried out in Cruz das Almas (State of Bahia, Brazil, 12°40′39″S, 39°06′23″W) from 2016 to 2019, with the number of replications varying from three replications (2017, 2018, and 2019) to four replications (2016) in a randomized block design. The plots consisted of two rows with 16 plants per plot. Planting was performed from May to July (during the rainy season). Spacings between rows and plants were 0.9 and 0.8 m, respectively. All trial management was performed, whenever necessary, in accordance with the technical recommendations and standard agricultural practices for cassava. For the IITA dataset, phenotypic information was collected from Cassavabase (see text footnote 1), the final file consisted of 590 clones evaluated in Ibadan, Nigeria, from 1998 to 2012; the number of replications was mostly four, but it ranged from two to six for some clones. The IITA dataset was composed of 77 trials in total with varied experimental designs. This dataset originated from a previous curatorship in which Ogbonna et al. (2021b) retrieved 228 trials out of 393 trials, and then we further filtered by location (Ibadan) and years (1998–2012). The total number of observations was 8,355 and 5,158 for Embrapa and IITA datasets, respectively (Table 1).

**TABLE 1:** Experimental areas, cities, geographical coordinates, years, number of phenotypical observations, and clones.

Institute	City	Geographical coordinates	Years	Observations	Clones
Embrapa	Cruz das Almas	12°40′42.4″S 39°05′27.8″W	2016–2019 (4 years total)	8,355	1,230
IITA	Ibadan	7°29′44.5″N 3°53′51.4″E	1998–2012 (15 years total)	5,158	590

For both datasets, HCN was measured according to the picrate titration method described by Fukuda et al. (2010), which is a qualitative determination of HCN content in fresh roots basis based on a 1–9 color scale; with 1 and 9 representing the extremes of low and high HCN, respectively. For the HCN Embrapa dataset, roots with uniform shapes and sizes from different plants that represent the plots were analyzed for HCN. Plants were harvested 11–12 months after planting. A cross-sectional 1 cm^3^ cut was made at the mid-root position, between the peel and the parenchyma center. The cube of root and five drops of toluene were added to a glass test tube, and they were tightly sealed with a stopper. In order to determine the qualitative score of HCN content, a strip of filter paper was dipped into a freshly prepared alkaline picrate mixture until saturation. Then, the saturated filter paper was placed above the root cube in the tube. Tubes were sealed for 10–12 h before the color intensity evaluation. HCN represents the total cyanogenic glucosides (HCN/CN^–^, linamarin and acetone cyanohydrin) in cassava root (Bradbury et al., 1999).

### Genotyping and Data Quality Control

For both Embrapa and IITA, the DNA was extracted from young leaves of cassava accessions according to a protocol described by Doyle and Doyle (1990). Then, the DNA was diluted in the TE buffer (10 mM Tris-HCl and 1 mM EDTA) and adjusted to a final concentration of 60 ng/μl. The DNA quality was checked by the digestion of 250 ng of genomic DNA from 10 random samples with the restriction enzyme *Eco*RI (New England Biolabs, Boston, MA, United States). Subsequent samples were sent to the laboratory for library preparation, sequencing, and bioinformatics analyses. Genotyping was performed by genotyping by sequencing (GBS) methodology, which is described in detail by Elshire et al. (2011). The DNA was digested with the *Ape*KI restriction enzyme and the Illumina sequencing read lengths of 150 bp. Marker genotypes were called with the TASSEL GBS pipeline V5 (Glaubitz et al., 2014) and aligned to the cassava reference genome version 6.1 (Illumina, San Diego, CA, United States). The joint dataset (Embrapa + IITA) was intersected using the GATK CombineVariants and Intersect functions (McKenna et al., 2010).

Filtering steps and parameters as well as imputation were performed as described in Ogbonna et al. (2021b). Filtering steps and parameters included: mean depth values > 5, missing data up to 0.2 per locus and minor allele frequency of 0.01 per loci, also, individuals with more than 0.8 of missing data per chromosome were removed. Imputation was performed for each chromosome using beagle version 4.1 (Browning and Browning, 2016). Imputed markers were subjected to further filtering using an allelic correlation greater or equal to 0.8. The final cassava clone set was obtained after filtering available phenotypic data on trial location and years basis, additional minor allele frequency filtering (0.05) step was carried out, achieving total sets with 1,230 clones and 14,323 SNPs for Embrapa, 590 clones, and 13,524 for IITA, 1,820 clones and 14,924 SNPs for the joint dataset (Embrapa + IITA) (Table 2) out of an intersected file with 4,814 clones. This number of SNPs seems to be suitable to capture or detect important QTL association for HCN based on the LD decay (r2 < 0.1) (Ogbonna et al., 2021a).

**TABLE 2:** Number of single nucleotide polymorphisms (SNP) in each data file after quality control, and the number of shared SNPs between data files after quality control^/1^.

Analyses	# SNPs
Embrapa	14,323
IITA	13,524
Embrapa+IITA	14,924
Common SNPs	11,883

*^/1^SNPs with minor allele frequency > 0.05 were kept.*

### Population Structure

The principal component analysis (PCA) and discriminant analysis of principal components (DAPC) analyses were performed with the joint dataset (Embrapa+IITA; 1820 clones; 14,924 SNPs), with the package “adegenet” (Jombart, 2008; Jombart et al., 2010) in R software (R Core Team, 2019). The PCA analysis and the principal components reported were based on the genomic kinship coefficients between clones. However, for DAPC analysis, the number of retained PCs (explained ∼ 90%) was based on the PCA of the marker matrix. Bayesian Information Criterion (BIC) was used to identify the optimal number of clusters. Complementary to DAPC and PCA, we also inferred population structure with STRUCTURE (Pritchard et al., 2000), selecting the 300 least correlated SNPs to warrant the use of unlinked markers. We tested the number of populations (K) varying from one to fifteen, running a series of ten independent runs for each value of K. Aiming the identification of the K number, 10,000 iterations were run, with 1,000 burn-in. For the choice of the most likely value of K, we used the ΔK method (Evanno et al., 2005) implemented in the Structure harvester software (Dent and Bridgett, 2012). After the identification of the K number, we ran a final analysis with 150,000 iterations, 50,000 burn-in, and the chosen K. Additional clustering step was carried out by Tocher’s method (Silva et al., 2017), grouping the DACP clusters based on their HCN means.

The fixation index (F_ST_) (Wright, 1965) between germplasm’s sources (Embrapa and IITA), as well as between clusters identified in the joint dataset’s DAPC analysis, was estimated by using the method of Weir and Cockerham (1984) with the package “hierfstat” (Goudet, 2005) in R software (R Core Team, 2019).

### Building the Haplotype Matrix

Single nucleotide polymorphisms (SNPs) obtained after quality control were used to build the haplotype blocks. Haplotypes were identified by Gabriel’s method (Gabriel et al., 2002), implemented on PLINK 1.90b5.3 (Purcell et al., 2007). The method is based on a confidence interval of DPrime (D′). The pairs of SNPs were considered in strong linkage disequilibrium (LD) if the upper boundary confidence interval of D′ was higher than 0.98 and the lower boundary was higher than 0.8. The maximum length of the blocks was set to 200 Kb. We assumed a very strong linkage between markers within the haplotype blocks. Then, the “haplotype matrix” was numbered (0 and 1) as it follows: for each haplotype block the class “0” was the one that contained exclusively 0 for all the haplotype blocks’ markers, whereas the class “1” consisted of all remaining haplotypes.

### Genomic Prediction Analyses

Clone’s GEBVs for HCN were predicted based on SNP effects estimated by the following analyses: (i) genomic prediction with training population of Embrapa genotypic and phenotypic data, (ii) genomic prediction with training population of IITA genotypic and phenotypic data, and (iii) genomic prediction with training population of the joint (Embrapa+IITA) datasets. The SNP effects were estimated in the training populations. Then, these three vectors of SNPs effects were multiplied by the genotype’s incidence codes of the mutual testing populations and summed to form the GEBVs.

The models used for the genomic prediction analyses by single markers and haplotypes were:


$$\begin{array}{c} y_{1}=X_{1}b_{1}+Z_{11}a_{G1}+Z_{21}a_{YR1}+{ɛ}_{1}\\ y_{2}=X_{2}b_{2}+Z_{12}a_{G2}+Z_{22}a_{YR2}+{ɛ}_{2}\\ y_{3}=X_{3}b_{3}+Z_{13}a_{G3}+Z_{23}a_{YR3}+{ɛ}_{3} \end{array}$$

Where, *y*_1_, *y*_2_, and *y*_3_ are the vectors of phenotypic observations and *b*_1_ and *b*_2_ are the scalars of mean fixed effect, for analyses of datasets of Embrapa and IITA separately, respectively, and *b*_3_ is the vector of fixed effects of the country added to the mean for the joint dataset’s analysis. The vectors *a*_*Gij*_ refers to the random additive genomic clone effects associated with the i-th dataset (*i* 1, 2, 3), *a*_*Gij*_ ∼ N(**0**, $\sigma_{a\,G\,i\,j}^{2}\,G_{i\,j}$), where *G*_*ij*_ is the additive genomic relationship matrices as defined below, $\sigma_{a\,G\,i\,j}^{2}$ is the genetic variance associated with *a*_*Gij*_. The random-effects vectors *a*_*YRi*_ refers to the combination of year and replication associated with the i-th dataset (*i* = 1, 2, 3), *a*_*YRi*_ ∼ N(**0**, $\sigma_{a\,Y\,R\,i}^{2}\,I_{y\,r\,i}$), where *I*_*yri*_ is an identity matrix and $\sigma_{a\,Y\,R\,i}^{2}$ is the variance estimate due to the combination of year and replication. *X*_*i*_, *Z*_*1i*_ and *Z*_*2i*_ are the incidence matrices for fixed effects and the random effects of genotypes and combination of year and replication, respectively, associated with the i-th dataset (*i* = 1, 2, 3). ε_*i*_ represents the residual vector associated with the i-th dataset (*i* = 1, 2, 3), ε_*i*_ ∼ N(**0**, $\sigma_{e\,i}^{2}\,I_{e\,i}$), where *I*_*ei*_ is an identity matrix and $\sigma_{e\,i}^{2}$ is the residual variance. Based on previous studies on the evaluation of different genomic selection models in cassava (Wolfe et al., 2017; Andrade et al., 2019), we have adopted genomic best linear unbiased prediction (GBLUP) as the standard prediction method. Therefore, the genomic predictions were based on the GBLUP method with the REML (restricted maximum likelihood) estimation of variance components.

The whole procedure uses the additive genomic relationship matrix *G*_*ij*_. Likelihood Ratio Tests (LRT) were undertaken to test the random effects using the chi-square distribution, with degrees of freedom equal to the difference in the number of parameters for the two models. Genomic prediction analyses were performed using the package “sommer” (Covarrubias-Pazaran, 2016) in R software (R Core Team, 2019).

Heritability was estimated as the ratio of the genetic variance to the sum of the genetic variance, the variance due to the combination of year and replication, and the residual variance, for each analysis.

To capture genetic relatedness, the genomic relationship matrix (*G*_*ij*_; with the i-th dataset (*i* = 1, 2, 3) and the j-th genotyping method (j = *SNP*, *hap*) for the genomic prediction of single markers and haplotypes were obtained as it follows:


$$\begin{array}{ccc} G_{i\_SNP} & = & \frac{{MM}^{\prime }}{\Sigma_{i=1}^{n}2piqi}\\ G_{i\_hap} & = & \frac{{HH}^{\prime }}{\Sigma_{i=1}^{n}piqi} \end{array}$$

Where, p_*i*_ and q_*i*_ are the allele frequencies for single markers’ analyses and the ‘allele’ frequencies for haplotypes’ analyses, **M** is the markers incidence matrix (numbered as 0, 1, and 2) centered by the mean 2p_*i*_ and **H** is the haplotypes incidence matrix (numbered as 0 and 1). The sizes of *G*_*ij*_ matrixes were 1,230 × 1,230, 590 × 590, and 1,820 × 1,820, for analyses (datasets) 1, 2, and 3, respectively. And the sizes of *I*_*yri*_ identity matrices for the combination of year and replication were 26 × 26, 60 × 60, and 86 × 86, for analyses 1, 2, and 3, respectively.

The SNP and haplotype effects vectors ($\hat{m},\hat{h}$) were calculated as it follows:


m^=(MM′)−1M′a^Gijh^=(HH′)−1H′a^Gij

The vector *a*_*Gij*_ refers to the random additive genetic effects *[a*_*Gij*_ ∼ N(**0**,$\sigma_{a\,G\,i\,j}^{2}\,G_{i\,j}$)] associated with the i-th dataset (*i* = 1, 2, 3) and the j-th genotyping method (j = *SNP*, *hap*). The GEBVs’ vectors were obtained as follows: GEBV_SNP^=Mm^ or GEBV_hap^=Hh^, for using SNPs or haplotypes, respectively.

For the cross-country analyses, simulating the hypothetical situation where we would not have the phenotypic information of a group of clones from a certain research institute/country, for the vector of GEBV prediction, we have used the estimated SNP effects with data from another research institute/country, after filtering the matrix **M** to encompass only common SNPs that were in both datasets.

Trait estimates of predictive ability, accuracy, and bias were calculated from cross-validation with the training sets for each analysis. Those parameters were estimated for each fold, and the value presented in this study is the mean of the folds. The cross-validation method used was the k-*fold*, k = 10, with the clones being randomly assigned to each fold. The training set, composed of 9 of the 10 subsets, was used to estimate marker effects and the remaining subset was the validation set. These marker effects estimates were used to predict the genomic breeding values of the validation set individuals. This process was repeated until all 10 subsets had been used as the validation population once.

The predictive ability was given by the correlation coefficient between predicted genetic values (GEBVs) and the phenotypes in the validation population. The accuracy was calculated as the ratio between the predictive ability and the square root of the phenotypic trait heritability. The phenotypes (PP) were linearly regressed on the GEBVs, and the regression coefficient $\hat{b}$_PP,GEBV_ was used to measure the degree of bias of the GEBV prediction. The bias relates to the size of the absolute differences between clones’ predicted genetic values and their pseudo-phenotypes. The estimated magnitude of these differences can be quantified by the $\hat{b}$_PP,GEBV_ regression coefficient and can be overestimated ($\hat{b}$_PP,GEBV_ < 1) or underestimated ($\hat{b}$_PP,GEBV_ > 1). A regression coefficient equal to one indicates no bias. Then, here we will represent bias as one unit minus the regression coefficient $\hat{b}$_PP,GEBV_ (Bias = 1 - $\hat{b}$_PP,GEBV_).

We compared the top (lower GEBVs for HCN) 100 ranking lists contrasting the predicted and estimated GEBVs for both Embrapa and IITA datasets (analysis 1 – TP with Embrapa’s dataset – and analysis 2 – TP with IITA’s dataset –, respectively), considering the hypothetical situation that in the absence of phenotypic characterization for one research institute/country, we might want to use the marker effects obtained from the GP using TP data from the other research institute/country to predict the performance of their clones.

Statistical analyses were based on the HCN phenotypes originated by the picrate method 1–9 color scale; however, for data visualization clone’s GEBV were further classified into three classes: sweet, intermedium, and bitter, as clones ranged from 1 to 4, 4.1 to 5, and 5.1 to 9, respectively.

## Results

### Population Structure

**FIGURE 1:**
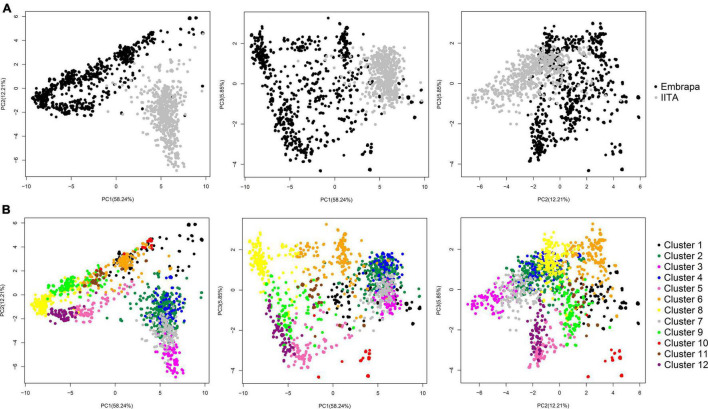
Principal components analysis (PCA) of the genomic kinship coefficients between cassava clones. **(A)** PCA from the genomic relationship matrix between all cassava clones (*N* = 1,820; 14,924 SNPs) from Brazil (Embrapa) and Nigeria (IITA), showing the first three principal components and the variance explained by each component in parenthesis on the corresponding axis (58.24, 12.21, and 5.85% for PC1, PC2, and PC3, respectively). In black representing Embrapa clone’s dispersion and in grey representing IITA’s. **(B)** PC diagram highlighting the 12 clusters identified by the DAPC analysis.

Principal components analysis in our joint dataset revealed a clear pattern of grouping clones according to their origin (Embrapa/Brazil and IITA/Nigeria), with the first three PCs accounting for 76.3% of the genetic variation (58.24, 12.21, and 5.85% for PC1, PC2, and PC3, respectively) (Figure 1A).

Similar to our PCA, in our first STRUCTURE run, two large groups were found. One is predominantly composed of clones from Embrapa (pop1 – 924 clones from Embrapa and 23 clones from IITA), and the other (pop2) with 567 (out of the total 590) clones from IITA in addition to 306 clones from Embrapa (Supplementary Figure 1). Additional STRUCTURE analyses were run within pop1 and pop2, separately. Pop1 and pop2 were subdivided into pop1a and pop1b and pop2a and pop2b, respectively. Pop2a were composed by Embrapa and IITA clones, almost half: half ratio, that is apparently closer related (274 clones from Embrapa and 302 clones from IITA), and pop2b were composed by almost exclusively IITA’s clones (32 clones from Embrapa and 265 clones from IITA; Supplementary Figure 2 and Supplementary Table 2). This shows that even though, at first, it looked like the datasets of the two research institutions consisted of two very distinct groups, there is certain intersectionality, and clones from different origins could be more admixed than clones from the same origin.

For DAPC, 500 PCs that explained ≈ 90% of the genetic variance were kept in our dataset. The optimal number of 12 genetic clusters was suggested to explain the total genetic variability based on BIC (Supplementary Figure 3), with cluster size ranging from 61 (cluster 10) to 299 individuals (cluster 2). Out of the 12 clusters, only four had clones from both Embrapa and IITA germplasm (clusters 1, 2, 6, and 7) with unbalanced proportions and a dominant origin within those clusters. The remaining eight clusters were exclusively composed of clones from Embrapa (clusters 5, 9, 10, 11, and 12) or IITA (clusters 3, 4, and 8). The HCN means ranged from 3.75 ± 0.68 (cluster 10) to 7.15 ± 0.84 (cluster 5) (Table 3). The principal components plot according to the clustering from DAPC analysis is shown in Figure 1B.

**TABLE 3:** Discriminant analysis of principal components (DAPC) that accounted for most (>95%) of the total genetic variability and genetic clusters were inferred (*k* = 12) based on Bayesian Information Criterion (BIC).

Cluster	N (Total)^/1^	n_1_ (Embrapa)	n_2_ (IITA)	Mean HCN^/2^
1	143	134	9	4.39 ± 0.94^a^
2	299	10	289	5.37 ± 0.84^b^
3	86	–	86	5.51 ± 0.89^b^
4	79	–	79	5.53 ± 0.79^b^
5	133	133	–	7.15 ± 0.84^c^
6	269	255	14	5.44 ± 1.38^b^
7	297	296	1	6.80 ± 0.97^c^
8	112	–	112	5.60 ± 1.02^b^
9	173	173	–	5.26 ± 1.37^b^
10	61	61	–	3.75 ± 0.68^d^
11	68	68	–	4.69 ± 1.37^a^
12	100	100	–	7.08 ± 0.71^c^
Total	1820	1230	590	5.57 ± 1.38

*Total number of clones (N), number of clones from Embrapa (n_1_), and number of clones in IITA (n_2_) in each cluster.*

*^/1^Size of the dataset used for the DAPC: 1820 clones and 14,924 SNPs.*

*^/2^Clustering by the Tocher method (Inter-cluster distance limit = 0.64).*

*^a,b,c,d^Mean clustering of the clusters by Tocher’s method.*

The intergroup distance limit was 0.64 with DACP clusters 5 (7.15 ± 0.84), 7 (6.8 ± 0.97), and 12 (7.08 ± 0.71) being allocated in the same Tocher group; high chances of the bitterest clones belonging to that group of clusters. They were exclusively composed of clones from Embrapa, except for cluster 7 which had 296 clones from Embrapa and only one from IITA. On the other hand, the ‘sweetest’ (low HCN) Tocher’s group was composed by cluster 10 (3.75 ± 0.68), only, exclusively composed of clones from Embrapa.

The F_ST_ values between the proposed mutual training and validation sets, composed of Embrapa and IITA’s genotypic datasets, were moderate (0.072). Estimated fixation indexes with markers between the twelve clusters identified within the joint dataset (Embrapa+IITA) varied from 0.002 (clusters 2 and 4, clusters 4 and 6) to 0.091 (clusters 3 and 9) (Table 4). Cluster 2 (mean HCN = 5.37 ± 0.84) was composed mainly of clones from IITA, while cluster 4 (mean HCN = 5.53 ± 0.79) was composed exclusively of clones from IITA, whereas cluster 6 (mean HCN = 5.44 ± 1.38) had mainly clones from Embrapa. Clusters 3 (mean HCN = 5.51 ± 0.89) and 9 (mean HCN = 5.26 ± 1.37) had clones exclusively from IITA and Embrapa, respectively. The above-mentioned clusters were all in the same Tocher’s group based on HCN mean.

**TABLE 4:** Fixation index (F_ST_) estimated by single nucleotide polymorphisms (SNPs) between cassava clones from Embrapa and IITA and between clusters identified within the joint dataset (Embrapa+IITA).

F_ST_ between genotypic data from Embrapa and IITA = 0.072											
Clusters	Clusters										
	2	3	4	5	6	7	8	9	10	11	12
1	0.004	0.013	0.007	0.005	0.009	0.018	0.084	0.084	0.074	0.023	0.030
2	0.000	0.004	0.002	0.009	0.003	0.019	0.086	0.086	0.073	0.019	0.026
3	0.004	0.000	0.003	0.020	0.005	0.023	0.090	0.091	0.076	0.020	0.024
4	0.002	0.003	0.000	0.011	0.002	0.018	0.087	0.086	0.072	0.020	0.023
5	0.009	0.020	0.011	0.000	0.013	0.023	0.090	0.090	0.082	0.030	0.037
6	0.003	0.005	0.002	0.013	0.000	0.018	0.084	0.083	0.070	0.023	0.024
7	0.019	0.023	0.018	0.023	0.018	0.000	0.032	0.030	0.023	0.007	0.009
8	0.086	0.090	0.087	0.090	0.084	0.032	0.000	0.004	0.023	0.043	0.047
9	0.086	0.091	0.086	0.090	0.083	0.030	0.004	0.000	0.020	0.040	0.043
10	0.073	0.076	0.072	0.082	0.070	0.023	0.023	0.020	0.000	0.027	0.027
11	0.019	0.020	0.020	0.030	0.023	0.007	0.043	0.040	0.027	0.000	0.008
12	0.026	0.024	0.023	0.037	0.024	0.009	0.047	0.043	0.027	0.008	0.000

**FIGURE 2:**
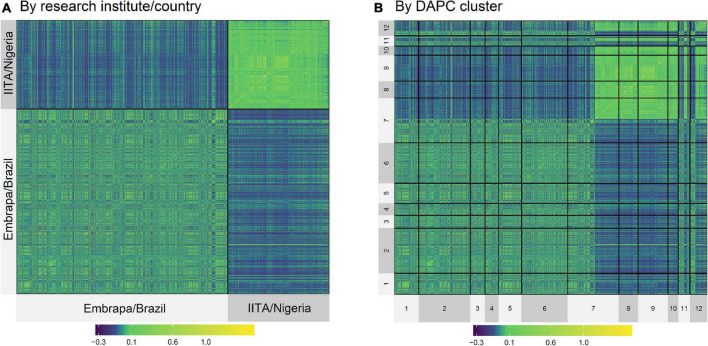
Heatmap of the kinship G matrix by Institute/Country **(A,B)** by DAPC Cluster (1–12).

Although the highest F_ST_ were expected to be among groups of clones from Embrapa and IITA, there were groups with clones exclusively from Embrapa (clusters 5 and 9) and exclusively from IITA (clusters 3 and 8) that presented moderate F_ST_ between them of 0.09, evidencing the genetic variability within the germplasms of each origin. In the same way, there were clusters from different origins that presented low F_ST_. The heatmap of the kinship matrix **G** also illustrated the population structure (Figure 2), highlighting the high coefficients between clusters 8 and 9 (F_ST_ = 0.004; Table 4), composed of clones from IITA and Embrapa, respectively.

Interestingly, when comparing DAPC and STRUCTURE clones’ grouping, the STRUCTURE group pop2a that was composed of 274 clones from Embrapa and 302 clones from IITA had mostly clones from DAPC groups 2 (277 out of group 2 total 290 clones – IITA), 5 (109 out of group 5 total 133 clones – Embrapa) and 12 (73 out of group 12 total 100 clones – Embrapa). DAPC groups 5 and 12 were groups with the highest HCN means (Table 3). These results are also in accordance with our estimated F_ST_ between DAPC groups, since F_ST_ between groups 2 and 5, 2 and 12, and 5 and 12 were 0.009, 0.026, and 0.037, respectively, representing very low population differentiation (Table 4).

### Genomic Analyses for Embrapa, International Institute of Tropical Agriculture, and Joint Datasets

All datasets had significant (*p* < 0.01) genetic and year-replication variability for HCN (Table 5). The phenotype-based heritabilities were 0.76, 0.18, and 0.56 for Embrapa, IITA, and the joint Embrapa-IITA datasets, respectively; while the SNP-based heritabilities were 0.97, 0.2, and 0.65 for Embrapa, IITA, and the joint Embrapa-IITA datasets, respectively. Embrapa’s dataset presented the highest mean and lowest coefficient of residual variation for HCN when compared to IITA and joint datasets.

**TABLE 5:** Estimates of phenotype-based heritability (H^2^), SNP-based heritability or haplotype-based heritability (h^2^), predicted mean, coefficient of residual variation (CVe), genetic variance ($\sigma_{G}^{2}$), variance due to the combination of year and replication ($\sigma_{Y\,R}^{2}$), residual variance ($\sigma_{\varepsilon }^{2}$), predictive ability (PA), accuracy (Ac) and bias for individual markers, and haplotypic block genomic analyses for hydrogen cyanide (HCN) content in Cassava.

**Single markers**										
**Datasets**	**H^2^**	**h^2^**	**Mean**	**CVe**	$\sigma_{G}^{2}$	$\sigma_{\mathrm{YR}}^{2}$	$\sigma_{\varepsilon }^{2}$	**PA**	**Ac**	**Bias^/2^**

Embrapa	0.76	0.97	7.11	0.13	31.90*^/1^	0.25*	0.79	0.59 ± 0.05	0.67 ± 0.06	0.41 ± 0.06
IITA	0.18	0.20	4.95	0.28	0.64*	0.62*	1.92	0.26 ± 0.12	0.61 ± 0.29	0.39 ± 0.37
Embrapa+IITA	0.56	0.65	5.57	0.21	3.68*	0.59*	1.39	0.64 ± 0.05	0.85 ± 0.07	0.23 ± 0.06

**Haplotype blocks**										

**Datasets**	**H^2^**	**h^2^**	**Mean**	**CVe**	$\sigma_{G}^{2}$	$\sigma_{Y\,R}^{2}$	$\sigma_{\varepsilon }^{2}$	**PA**	**Ac**	**Bias**

Embrapa	0.76	0.96	5.91	0.16	31.00*	0.27*	0.86	0.48 ± 0.07	0.56 ± 0.08	0.58 ± 0.08
IITA	0.18	0.20	5.05	0.27	0.65*	0.62*	1.92	0.22 ± 0.14	0.51 ± 0.34	0.48 ± 0.38
Embrapa+IITA	0.56	0.62	5.74	0.21	3.31*	0.59*	1.42	0.60 ± 0.04	0.80 ± 0.05	0.30 ± 0.05

^/1^ *Significant at 0.01 by the likelihood ratio test (LRT). LRT = 2*(complete model log-likelihood – reduced model log-likelihood). ^/2^Bias = 1 – $\hat{b}$_PP,GEBV_.

For the single markers’ analyses, the highest predictive ability, accuracy, and the lowest bias were achieved when using the joint dataset, being 0.64 ± 0.05, 0.85 ± 0.07, and 0.23 ± 0.06, respectively. For Embrapa and IITA datasets alone, the SNP-based predictive abilities were 0.59 ± 0.05 and 0.26 ± 0.12, the accuracies were 0.67 ± 0.06 and 0.61 ± 0.29 and the biases were 0.41 ± 0.06 and 0.39 ± 0.37, respectively. For all genetic parameters, the highest standard deviations of 10-folds were found when using the IITA dataset.

**FIGURE 3:**
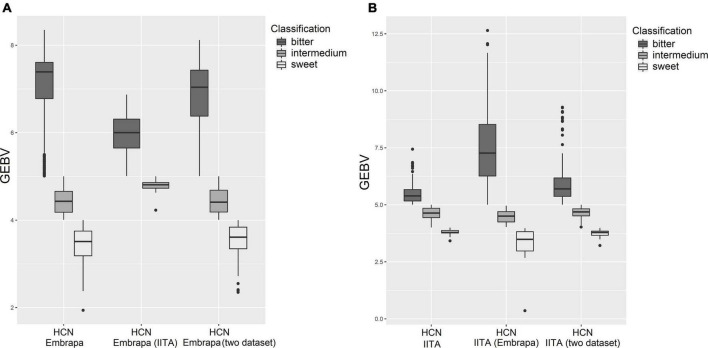
Boxplots contrasting the hydrogen cyanide predictions on country-dataset and cross-country-dataset to show sweet, intermedium and bitter classes. **(A)** Hydrogen cyanide for Embrapa’s clones based on GEBV prediction on Embrapa’s dataset, IITA’s dataset and the two datasets together, respectively. **(B)** Hydrogen cyanide for IITA’s clones based on GEBV prediction on IITA’s dataset, Embrapa’s datasets and the two datasets together, respectively.

From the practical point of view, for HCN there is still a reclassification, group-allocating according to the picrate method (1–9)’s records, which are: ranging from 1 to 4 - Sweet; from 4.1 to 5 – Intermedium; from 5.1 to 9 – Bitter. Figure 3 illustrates boxplots contrasting the HCN estimated GEBVs (predicted by single markers) on the country-specific dataset, cross-country-dataset and considering both datasets jointly to characterize sweet, intermedium, and bitter classes for Embrapa and IITA germplasm. For the GEBVs of clones from IITA predicted with the Embrapa dataset, the predicted values exceeded the sample space limits of the picrate method (1–9). It seems that cross-country genomic predictions, in the present work, underestimated the GEBVs for Embrapa clones predicted by marker effects from IITA’s dataset GP and overestimated the GEBVs for IITA clones predicted by markers effects from Embrapa’s dataset GP.

### Genomic Analyses With Single Markers vs. Haplotypes

For Embrapa, IITA, and the joint datasets, 3,132, 2,678, and 3,337 haplotype blocks were found with mean lengths (Kb) of 20.4, 33.189, and 19.636 Kb and mean number of SNPs of 3.616, 4.086, and 3.569 SNPs, respectively (Supplementary Table 1).

Although we have assumed a simplified approach to number the ‘haplotype matrix’, for the haplotype genomic prediction analyses (Table 5), the accuracy recovering rates with haplotype blocks analyses compared to single markers were high: 84% (0.56/0.67), 84% (0.51/0.61), and 94% (0.8/0.85), for the three datasets. Predictions from haplotype blocks had lower accuracies and higher biases compared to single markers; however, the correlation between the GEBVs estimated by the analysis of single markers and haplotype blocks were 0.99, 0.95, and 0.99 for Embrapa, IITA, and the joint dataset, respectively. Model complexity reductions (haplotypes number/single markers number) from using haplotypes were down to 22% (3,132/14,323), 20% (2,678/13,524), and 22% (3,337/14,924) of the original complexity, for Embrapa, IITA, and the joint dataset, respectively.

### Comparisons Between Genomic Estimated Breeding Values Rankings

For Embrapa clones’ GEBVs estimated by markers effects in the three analyses (analysis 1: Embrapa phenotypic and genotypic data, analysis 2: IITA phenotypic and genotypic data, and analysis 3: the two datasets together), high correspondences between GEBVs estimated by analyses 1 and 2, 1 and 3, and 2 and 3 (Pearson’s correlations of 0.55, 0.96, and 0.6, respectively, whereas for Kendall’s correlations they were 0.38, 0.82, and 0.43, respectively) were identified. For IITA clones’ GEBVs estimated by markers effects in the three analyses, weak but positive correspondences were obtained between GEBVs estimated by analyses 1 and 2, and 1 and 3 (Pearson’s correlations of 0.1 and 0.15, respectively, whereas for Kendall’s correlations they were 0.07 (1 vs. 2), 0.1 (1 vs. 3), and 0.78 (2 vs. 3), respectively). For analyses 2 and 3 a good correspondence was observed (correlations of 0.92).

We assessed the correlation between the common SNP’s effects vectors (total of 11,883 SNPs) originated from the three analyses. For SNP effects from analyses 1 and 2 the correlation was 0.01, from analyses 1 and 3, and 2 and 3, they were 0.69 and 0.42, respectively.

We also compared the top (lower GEBVs for HCN) 100 clones ranking contrasting the predicted and estimated GEBVs for both Embrapa and IITA datasets (analyses 1 and 2, respectively), considering the hypothetical situation that in the absence of phenotypic characterization for one research institute/country, we might want to use the marker effects obtained from the GP using data from the other research institute/country to predict the GEBVs’ performance of their clones. For Embrapa, from the top 100-lists’ comparison, we have found 12 coincident clones (Supplementary Table 3), while for IITA lists we have found 24 coincident clones (Supplementary Table 4). Out of the coincident clones, those from Embrapa are in DAPC clusters of 1 and 10 and those from IITA are in DAPC clusters of 1, 2, 4, 6, and 8. From these clusters, 1, 2, and 6 are mixed, while 4, 8, and 10 are composed exclusively by IITA or Embrapa clones. Supplementary Figure 4 illustrated where those coincident clones between own- and cross-country genomic predictions fall in the PCA plot.

## Discussion

### Population Structure

This study was conceptualized as a proof-of-concept to assess the feasibility of using cross-country genomic predictions to support the selection of the most suitable clones for germplasm exchange. Moreover, the germplasm characterization for preemptive breeding purposes, in case of quarantine diseases, i.e., when we might not have the phenotypes (resistance to certain disease because the screening in a greenhouse or the field is forbidden) is another important area that can benefit from the results of the present study. In such a case, the estimation of the clones’ GEBVs by using markers’ effects estimated in different training populations can be used to rank the most promising clones with resistance to the disease. However, it is well known the population structure has a strong effect on genomic prediction estimates, as observed in both animals (Hayes et al., 2009a) and plants (Technow et al., 2013).

Understanding the population structure of the joint dataset, how the cassava clones from Embrapa-Brazil and IITA-Nigeria are related to each other, is important in order to better plan the germplasm exchange dynamic between Brazil and Nigeria, or even to understand the outcomes from the joint dataset and the mutual cross-country predictions. Guo et al. (2014) investigated the impacts of population structure on the evaluation of genomic heritability and found that population structure explained 33 and 7.5% of genomic heritability for rice and maize, respectively, depending on the trait tested, with within-subpopulation variation explaining the remaining heritability. The sample size, the observed variability among populations, and traits require resource optimization that incorporates knowledge about the parameters’ variability (Riedelsheimer et al., 2013).

Population structure exists due to geography, history of domestication and genetic background, natural or artificial selection. In general, our results, obtained by different methods to assess genetic differentiation between populations (PCA, DAPC, F_ST_, and STRUCTURE), point to the same common point: there is clearly a population structure between the germplasm from Embrapa (Brazil) and the IITA’s (Nigeria), however, this differentiation is not strong enough that there are no clones between these research programs that are more similar than divergent clones within the own program itself. It may be related to past clone exchange (Latin America and Africa), mislabeling, among others. It is important to highlight that DAPC and STRUCTURE are *ad hoc* methods, then, likely (and even expected) to present different results in terms of clustering. Nevertheless, their results can be complementary. As they were in the present study, which generates speculation and perspective for future studies about the reason of the groups of clones from Embrapa with the highest content of HCN (DAPC groups 5 and 12) are those that are apparently more genetically related to a considerable part of the clones from IITA (group 2), and their respective STRUCTURE pop2a including Embrapa and IITA clones. Future studies with dry matter content and fresh root yield could shed light on this issue, as these traits appear to be more decisive in driving artificial selection over the years.

The fixation index (F_ST_) between the proposed mutual training and validation sets, composed by Embrapa and IITA’s germplasm, were moderate (0.072), representing a weak population differentiation. When studying the population structure of Brazilian germplasm from Embrapa separately, Ogbonna et al. (2021b) found almost the same value, specifically an overall pairwise F_ST_ of 0.073 in 10 clusters obtained by DACP. The predictive ability was higher by using the joint dataset (Embrapa + IITA) (0.64) than using the IITA population due to the training set sizes (1,820 for both and 590 for IITA). However, the Embrapa population showed almost the same predictive ability of the joint dataset (0.59 and 0.64, respectively), despite the difference in the training set sizes (1,230 individuals in Embrapa and 1,820 individuals in the joint dataset). Consequently, the use of the Embrapa population to predict IITA genotypes is feasible, which had already been evidenced by the moderate fixation index (0.072). Scutari et al. (2016) found that the correlation between true and predicted values decays approximately linearly with respect to the fixation index between the training and the target populations. If more phenotyped individuals were included in IITA’s training set, the higher predictive ability would be expected by using the IITA population to predict Embrapa’s. Noteworthy, if we had a combination of higher F_ST_ and lower predictive ability than the achieved in the present study, the use of any single population to predict another would be impractical and could compromise accurate GEBV prediction. Hence, it would be mandatory for the inclusion of individuals from other populations for GEBVs prediction and success of these breeding programs’ consortia (de Roos et al., 2009).

To provide the comprehensive genetic architecture of HCN in cassava, Ogbonna et al. (2021a) performed a GWAS with a Brazilian dataset and validated it in an African population and a joint GWAS analysis between Africa and Brazil. These authors also showed evidence that the genetic architecture of HCN is conserved between the two continents. Ogbonna et al. (2021a) study revealed HCN is regulated in an oligogenic manner with two major loci explaining the variation across their datasets. Wolfe et al. (2019) identified regions in the genome of cultivated cassava (*Manihot esculenta*) in Africa that had introgressions of *Manihot glaziovii*, the legacy of crosses during the 1930s to improve varieties and mitigate the effects of emerging plant diseases. Although crosses with *Manihot glaziovii* were not as frequent in Brazil as they were in Africa at that time, recently Ogbonna et al. (2021b) found introgressions of *M. glaziovii* in the Brazilian germplasm as well (on chromosomes 5 and 17, while those reported in African germplasm were on chromosomes 1 and 4).

### Genomic Analyses for Embrapa, International Institute of Tropical Agriculture, and Joint Datasets

Comparing the dataset’s genetic parameters estimates, Embrapa’s dataset yielded the largest genetic variance estimates for HCN compared to IITA, whereas the latter had the largest estimates for year-replication and residual variance estimates. The highest mean for HCN were observed for the Embrapa dataset. Ogbonna et al. (2021a) explored the distribution of cyanide across sub-Saharan Africa datasets, by leveraging open-source data; their analysis indicated that Central and Southern Africa showed on average higher cyanide varieties compared to West Africa, and they also revealed a very slight trend of lowering cyanide on landraces compared to improved varieties.

The observed differences between heritability estimates from Embrapa-Brazil and IITA-Nigeria germplasm could be attributed to several factors, e.g., environmental influence, genetically related plants, phenotypic measurement errors, imputation, and could have been potentialized by germplasm’s mislabeling. Clone mislabeling has been reported to reduce genetic gain by up to 40% in Africa (Yabe et al., 2018). Wolfe et al. (2017) reported varied heritabilities between populations, they discussed that it is conceivably as a consequence of differences in the number and experimental design of field trials among breeding programs, they also highlighted how is difficult to determine the reason for those differences in heritabilities between breeding programs for most of the traits.

In our study, pooling the two datasets together improved HCN predictive ability, accuracy as well as reducing the bias. Multi-population TP combining breeding populations can be employed to assess the potential for broadly focused GS training approaches (Faville et al., 2018). Multi-group training sets have already shown good predictive ability for animals (Pryce et al., 2011) and plants (Technow et al., 2013). In pigs, Song et al. (2019) reported the GP using combined populations with different genetic backgrounds or from different breeds has not shown a clear advantage over using within-population or within-breed GPs. Wolfe et al. (2017) reported that cross-population accuracy was generally low, with a mean of 0.18, but for prediction of cassava mosaic disease it has increased up to 0.57 in one Nigerian population when data from another related population were combined. According to Somo et al. (2020), adding the two cassava populations together to increase the TP size did not improve trait prediction accuracy. They evaluated cross-location predictions and compared the trait prediction accuracy estimates with the within-location prediction accuracy estimates. They reported that, from their nine traits evaluated, except for mean cassava green mite severity, the cross-location predictions were generally low, averaging between 0.10 and 0.14 when the Kibaha (Tanzania) population was used to predict the Ukiriguru (Tanzania) set and vice versa, respectively. The authors also included a Ugandan TP in either population, but that did not improve trait prediction accuracy either. In animal breeding, for cross-breed prediction, this limitation has been reported to be due to the non-persistent association between SNPs and QTL across breeds (Hayes et al., 2009b).

Future studies including clones that were evaluated at both IITA and Embrapa would be beneficial to assess cross-country genomic prediction in light of genotype-by-environment interaction. Another topic of interest that could be explored in future studies would be genomic predictions considering only the STRUCTURE pop2a (with a total of 576 clones; 274 from Embrapa and 302 from IITA) and to verify whether the correlations between GEBV vectors would be greater than those found in the present study (0.55 for prediction of Embrapa clones and 0.10 for prediction of IITA clones). However, restricting the most closely related groups would limit genetic variation (which did not appear to be a problem in our original proposal as we obtained satisfactory values for accuracy and predictive ability) and that could substantially alter the genetic parameters. It is worthy to mention that pooling together datasets is expected to increase precision and accuracy, due to an increase in sample size; by its turn, cross-population/location predictions tend to decrease them, due to change in the target. That was one of the criteria we took into account to narrow down multilocation datasets to a dataset strictly focused on Ibadan in our case. Those referred increase and decrease are in comparison with the within-population prediction. Whatever strategy is adopted, it is essential to maintain sufficient genetic variation in training populations in practical genomic selection (Guo et al., 2014).

Boxplots of estimated GEBVs group-allocated (sweet, intermedium, and bitter) on own country-dataset, cross-country-dataset and considering both datasets jointly (Figure 3) showed that, in the present work, cross-country genomic predictions seemed to have underestimated the GEBVs for Embrapa clones predicted by markers effects from IITA’s dataset GP and overestimated the GEBVs for IITA clones predicted by markers effects from Embrapa’s dataset GP. This could possibly be due in part to the difference between the mean magnitude for the Embrapa and IITA datasets. Therefore, focusing on the distribution and range of classes would also be important. The number of clones allocated to each class of HCN content, by the research institute and their considered analysis, is presented in Table 6.

**TABLE 6:** The number of cassava clones allocated in each class of HCN content, for Embrapa and IITA, according to the GEBVs estimated with marker effects from the three referred analyses.

Institute	Embrapa			IITA		
Classification^/1^	Analysis 1^/2^	Analysis 2	Analysis 3	Analysis 1	Analysis 2	Analysis 3
Sweet	263	0	243	18	36	22
Intermedium	165	79	187	260	72	171
Bitter	802	1151	800	312	482	397
Total clones	1230	1230	1230	590	590	590

*^/1^Classification according to the picrate method scale: ranging from 1 to 4 – sweet; from 4.1 to 5 – intermedium; from 5.1 to 9 – bitter. ^/2^Analysis 1, 2, and 3: marker effects’ prediction based with training datasets of Embrapa, IITA, and Embrapa+IITA, respectively.*

### Genomic Analyses With Single Markers vs. Haplotypes

The haplotype GP for HCN was an attempt to better capture the genomic similarity between lines due to the increased LD between haplotypes and causal genetic variants as well as to capture local allelic interactions. Although using haplotypes is often expected to improve the GP’s accuracy over single SNPs, in the present study it was not observed. Lower predictive ability and accuracies were found by haplotype-based GP compared to single marker GP. Sallam et al. (2020) found, for *k*-fold cross-validation in wheat yield, that using haplotypes of 5, 10, 15, and 20 adjacent markers increased in 6.3, 2.9, 5.3, and 2.2%, respectively in the predictive ability over single markers. The authors pointed a trade-off regarding the haplotype length: on one hand, the increase of haplotype length is expected to capture LD between markers in blocks with QTL, thereby increasing the accuracy of prediction, on the other hand, this may also increase the number of haplotype “allelic” classes, which may reduce the accuracy of prediction due to smaller sample sizes representing these classes. The simplified approach proposed to build the haplotype’s matrix, could possibly explain why we observed slightly lower accuracies: our haplotypes were generated by Gabriel’s method (Gabriel et al., 2002) implemented on PLINK, and length set to maximum 200 Kb. Thus, the mean number of SNPs were of 3–4 SNPs spanning segment-length averaging of 20–33 Kb. However, the predicted GEBVs between the two approaches (haplotype vs. single markers) were highly correlated (>0.95).

A study performed by Ogbonna et al. (2021a) revealed that HCN is regulated in an oligogenic manner with a few major genomic regions explaining a great proportion of the variation. Our efforts on using haplotype blocks are aligned with the previous findings; the search for a simplified structure that can explain the behavior of the trait and predictability of clone performance across countries. The accuracy recovering rates with haplotype blocks analyses compared to single markers we have got from using only around 3,000 haplotypes is very interesting and appealing. Even with lower accuracy when compared to single marker GP, the results of haplotype-based GP were good enough especially taking into account details that may have limited the success of the haplotype approach in the present work: the low depth nature of GBS and its implication in the heterozygote’s SNP calling, and, also, the oligogenic architecture of HCN, which might have an impact on haplotypic definition and, consequently, on subsequent predictions. Thinking in the cassava context, handling of heterozygotes site through haplotype definition is an interesting strategy. These haplotypes are likely to encompass polygenic blocks with coadapted genes controlling important quantitative traits. This capturing of the important causal genetic variants and the LD between markers and QTL in blocks enables increasing the accuracy. These genes in blocks are relevant for both local variation (and adaptation) and stability (predictability) when they are genetically conserved across locations.

### Comparisons Between Genomic Estimated Breeding Values Rankings

One location’s data can be used to predict performance in another location, which can be helpful in accelerating the breeding process. However, according to Rio et al. (2020), when targeting a group-specific predicted set, training a model on a different group can eventually decrease accuracy as shown in several species.

For Embrapa clones, a correlation between GEBVs predicted with TP from Embrapa and IITA was 0.55, whereas for IITA clones, it was 0.1. For Embrapa, from the 100-lists comparison, we have found 12 coincident clones, while for IITA lists we have found 24 coincident clones, which lead us to think that although the observed correspondence between GEBVs vectors for IITA using marker effects from analyses 1 and 2 was weak (correlation of 0.10 between the vectors of estimated GEBVs), they could be enough to at least give some clue in clonal selection for germplasm exchange with much better probabilities than a blind-scenario, where it is not feasible to send all the clones available, and specifically when many clones demonstrate good performance for important agronomic traits.

It seems reasonable to attribute part of the success of these coincidences between the predictions of GEBVs obtained with the effects of markers from the country-specific analysis vs. from other country’s analysis to the similarities the regions (Cruz das Almas-Brazil and Ibadan-Nigeria) in terms of temperature and rainfall distribution. Both are in a low latitude range, belonging to tropical latitude zones; according to Köppen-Geiger Climate Classification, Cruz das Almas and Ibadan are lying into tropical rainforest climate (Af) and tropical savanna, wet (Aw), respectively. Other interesting variables to be mentioned: Cruz das Almas’ altitude of 224 m above sea level, 1,136 mm annual precipitation, and an average temperature of 23^°^C (within a range of 18–34^°^C), whereas Ibadan’s altitude of 199 m above sea level, 1,311 mm annual precipitation and an average temperature of 26.5^°^C (within a range of 20–34^°^C)^2^.

The poor prediction result for cross-program prediction reported here, using Embrapa’s TP to predict IITA clones’ GEBV (0.10) makes it a bit hard to conceive the cross-program predictions to be adopted in the daily routine of the referred breeding programs. However, in the lack of a better strategy, especially for the case of exchanging material in the context of quarantine diseases, which is a more delicate scenario with an existing restriction on clone movement, information at the molecular level could aid to the understanding of populations structure, diversity, and cross-country genomic predictions could corroborate to guide a germplasm selection to exchange by a reasonably accurate selection of clones since unlimited shipping would be unfeasible.

Nevertheless, when using IITA’s TP to predict Embrapa clones’ GEBV a correlation of 0.55 was observed which is of relevance for such a study. This discrepancy within the mutual cross-country GP may perhaps have been influenced by experimental technical details since the same trait showed so disparate heritabilities and coefficients of variation when evaluated in different research institutes/countries. From the GWAS results for HCN obtained by Ogbonna et al. (2021a) on IITA’s historical data, the error variances were higher compared to Embrapa’s as well. IITA’s dataset spans over more than a decade of experimental work which could certainly be influenced by changes in human resources, management, not to mention year variance which is larger. Thus, the potential impacts on prediction accuracy can change the ranking of top-performing clones in the validation population. Although it seems to have worked reasonably well in the present scenario, to make a conclusive statement regarding the use of cross-country predictions in cassava, further evidence is needed. This seems to be among the first attempts to evaluate the cross-country genomic selection in cassava and plants. As such, a lot of useful new information was provided on the subject, which can guide new research on this very important and emerging field.

## Conclusion

Twelve clusters were revealed by the assessment of population structure in South-American and African cassava. The fixation index among the clusters identified within the joint dataset ranged from 0.002 to 0.091. The joint dataset (Embrapa+IITA) provided an improved accuracy compared to the prediction accuracy of either germplasm’s sources individually. When using the marker effects estimated with IITA dataset as the training to predict the Embrapa clones’ GEBVs, we could say it was a successful case of cross-country prediction, taking into consideration the medium-to-high correlation of 0.55 between their vectors of estimated GEBVs; regardless, the reverse presented a low correlation of 0.1. Genomic predictions from haplotype blocks had slightly lower accuracies and higher biases compared to single markers. The correlation between the GEBVs estimated by the analyses of single markers and by haplotype blocks varied from 0.95 to 0.99, with high accuracy recovering rates of the haplotype blocks compared to single markers, ranging from 0.84 to 0.94. Also, with the haplotype blocks analyses, the model sizes were reduced, with complexity reduction rates going down to 0.2–0.22. Cross-country genomic predictions proved to have potential use under the present study’s scenario, i.e., investigated trait heritabilities higher than 0.18 and conserved genetic architecture across locations, with clones from regions that are not too much divergent in terms of meteorological conditions and with at least a minimal historical germplasm exchange.

## Data Availability Statement

The datasets presented in this study can be found in online repositories. The names of the repository/repositories and accession number(s) can be found below: ftp://ftp.cassavabase.org/manuscripts/Torres_et_al_2021/, Cassavabase.

## Author Contributions

LT, EO, and MR contributed to the interpretation of results and drafting of the manuscript. LM, CA, FF, and MR contributed to the brainstorming and critical review of this manuscript. EO, LT, AO, GB, and MR contributed to the conceptualization, data curation, formal analysis, investigation, methodology, and writing—original draft preparation. EO and MR contributed to project administration, supervision, and writing—review and editing. LT, AO, LM, and GB contributed to the data resources, curation, and writing—review and editing. GS contributed to data analysis. All authors contributed to the article and approved the submitted version.

## Conflict of Interest

The authors declare that the research was conducted in the absence of any commercial or financial relationships that could be construed as a potential conflict of interest.

## Publisher’s Note

All claims expressed in this article are solely those of the authors and do not necessarily represent those of their affiliated organizations, or those of the publisher, the editors and the reviewers. Any product that may be evaluated in this article, or claim that may be made by its manufacturer, is not guaranteed or endorsed by the publisher.

## Footnotes

1
https://cassavabase.org/

2
en.climate-data.org
