# Supplementary material for: Can Cross-Country Genomic Predictions Be a Reasonable Strategy to Support Germplasm Exchange? – A Case Study With Hydrogen Cyanide in Cassava
Source: Front Plant Sci. 2021 Dec 8;12:742638. doi: 10.3389/fpls.2021.742638 (PMC8692580; doi:10.3389/fpls.2021.742638)
Supplement: Supplementary file 1 [file Data_Sheet_1.docx]

Can cross-country genomic predictions be a reasonable strategy to support germplasm exchange? – A case study with hydrogen cyanide in cassava

**Supplementary material**

**Supplementary Table 1.** Number, maximum, mean and minimum length of haplotype blocks and number of SNPs within each one. Results from three datasets.

| Datasets | # Haplotype Blocks | Length (Kb) | | |  | # SNPs | | |
| --- | --- | --- | --- | --- | --- | --- | --- | --- |
|  |  | Max.^/1^ | Mean | Min. |  | Max. | Mean | Min. |
| Embrapa | 3,132 | 199.541 | 20.400 | 0.002 |  | 26 | 3.616 | 2 |
| IITA | 2,678 | 199.636 | 33.189 | 0.002 |  | 22 | 4.086 | 2 |
| Embrapa+IITA | 3,337 | 199.541 | 19.636 | 0.002 |  | 20 | 3.569 | 2 |

^/1^ Maximum length set to 200 Kb.

**Supplementary Table 2.** Number of clones from each breeding program in groups revealed by STRUCTURE analyses.

|  | Clones  pop1a | Clones  pop1b | Clones  pop2a | Clones  pop2b | Clones  total |
| --- | --- | --- | --- | --- | --- |
| Embrapa | 344 | 580 | 274 | 32 | 1,230 |
| IITA | 12 | 11 | 302 | 265 | 590 |
| Total | 356 | 591 | 576 | 297 | 1,820 |

**Supplementary Table 3.** Coincident twelve clones from the ‘top 100 clone’s list’ for Embrapa germplasm.

| Clone | GEBV1_1^/1^ | | GEBV1_2^/2^ | |
| --- | --- | --- | --- | --- |
| CNPMF1317.250372086 | 3.22 | (sweet) | 5.03 | (interm.) |
| CNPMF1332.250372041 | 3.10 | (sweet) | 5.03 | (interm.) |
| CNPMF1414.250372209 | 2.38 | (sweet) | 5.00 | (interm.) |
| CNPMF1444.250372212 | 2.53 | (sweet) | 4.99 | (interm.) |
| CNPMF1446.250372223 | 3.17 | (sweet) | 5.03 | (interm.) |
| CNPMF1507.250372194 | 2.58 | (sweet) | 5.03 | (interm.) |
| CNPMF1579.250407031 | 2.83 | (sweet) | 5.02 | (interm.) |
| CNPMF187.250370458 | 3.12 | (sweet) | 4.88 | (interm.) |
| CNPMF212.250370447 | 3.18 | (sweet) | 4.68 | (interm.) |
| CNPMF213.250370448 | 3.28 | (sweet) | 4.70 | (interm.) |
| CNPMF286.250370544 | 2.63 | (sweet) | 4.74 | (interm.) |
| Rosinha365.250437688 | 3.14 | (sweet) | 5.02 | (interm.) |

^/1^ GEBV1 = GEBVs estimated for EMBRAPA clones by genomic prediction with EMBRAPA phenotypic and genotypic data; ^/2^ GEBV1_2 = GEBVs estimated for EMBRAPA clones via SNPs’ effect estimated by genomic prediction with IITA phenotypic and genotypic data.

**Supplementary Table 4.** Coincident twenty-four clones from the ‘top 100 clone’s list’ for IITA germplasm.

| Clone | GEBV2_2^/1^ | | GEBV2_1^/2^ | |
| --- | --- | --- | --- | --- |
| I000378.250300406 | 4.03 | (interm.) | 4.54 | (interm.) |
| I030060.250303093 | 4.06 | (interm.) | 4.08 | (interm.) |
| I030061.250300215 | 4.45 | (interm.) | 4.83 | (interm.) |
| I030141.250300223 | 4.04 | (interm.) | 4.82 | (interm.) |
| I030256.250300226 | 4.13 | (interm.) | 4.45 | (interm.) |
| I062116.250304713 | 4.20 | (interm.) | 4.51 | (interm.) |
| I101014.250099332 | 3.87 | (sweet) | 0.36 | (sweet) |
| I101573.250399860 | 4.17 | (interm.) | 4.08 | (interm.) |
| I60142.250304629 | 4.24 | (interm.) | 4.08 | (interm.) |
| I920325.250304478 | 4.00 | (sweet) | 4.24 | (interm.) |
| I970299.250300471 | 4.18 | (interm.) | 4.25 | (interm.) |
| TMEB117.250304093 | 4.16 | (interm.) | 3.45 | (sweet) |
| TMEB199.250253760 | 3.80 | (sweet) | 3.61 | (sweet) |
| TMEB225.250253783 | 3.77 | (sweet) | 4.22 | (interm.) |
| TMEB239.250253789 | 4.16 | (interm.) | 4.78 | (interm.) |
| TMEB270.250253800 | 4.34 | (interm.) | 4.51 | (interm.) |
| TMEB403.250253858 | 4.32 | (interm.) | 3.51 | (sweet) |
| TMEB435.250253875 | 3.75 | (sweet) | 4.07 | (interm.) |
| TMEB455.250253885 | 3.76 | (sweet) | 4.09 | (interm.) |
| TMEB459.250253886 | 4.21 | (interm.) | 2.94 | (sweet) |
| TMEB469.250253889 | 4.36 | (interm.) | 3.75 | (sweet) |
| TMEB477.250253892 | 3.77 | (sweet) | 4.07 | (interm.) |
| TMEB480.250253894 | 3.79 | (sweet) | 4.25 | (interm.) |
| TMEB785.250254011 | 4.51 | (interm.) | 3.76 | (sweet) |

^/1^ GEBV2_2 = GEBVs estimated for IITA clones by genomic prediction with IITA phenotypic and genotypic data; ^/2^ GEBV2_1 = GEBVs estimated for IITA clones via SNPs’ effect estimated by genomic prediction with EMBRAPA phenotypic and genotypic data.

**
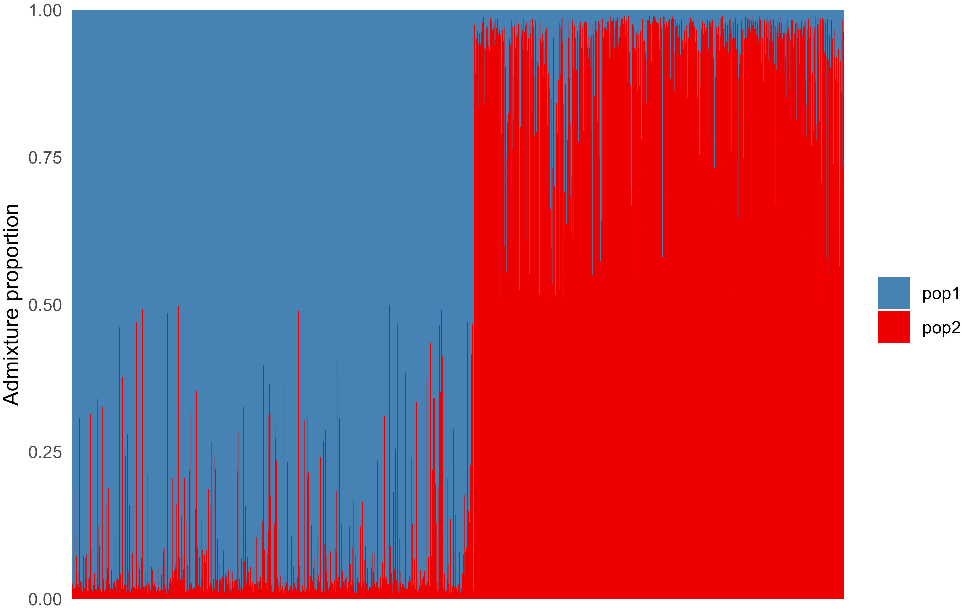
**

**Supplementary Fig 1.** Genetic relationships between the Embrapa and IITA populations (joint analyses) surveyed using STRUCTURE based on the 300 least correlated SNPs. The models with *K* = 2 were optimal based on the delta K value and the highest log-likelihood value, respectively.


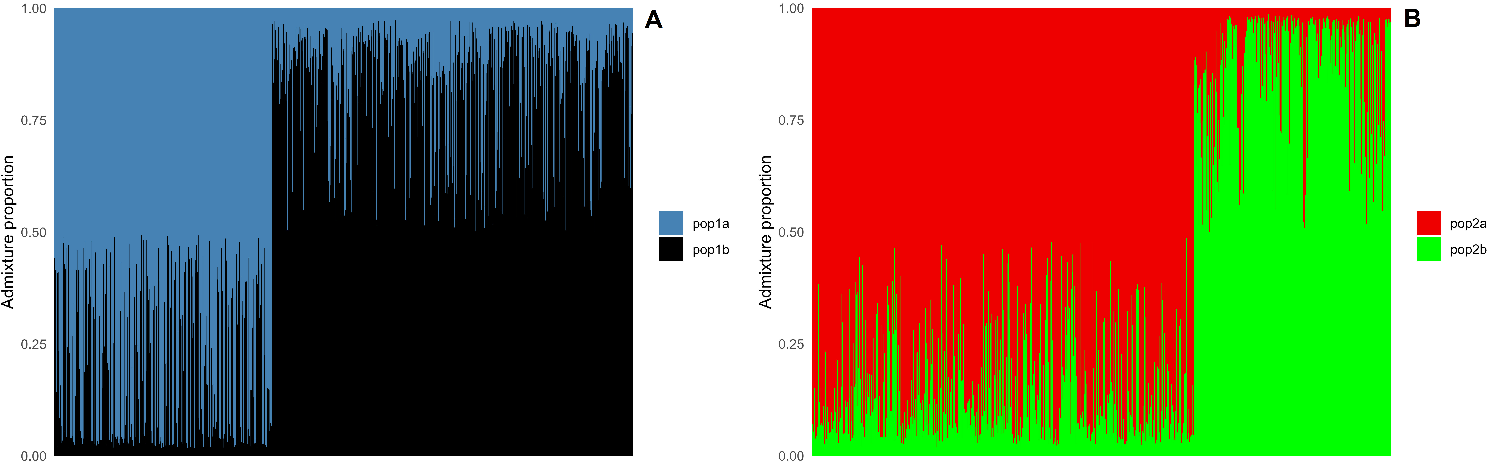


**Supplementary Fig 2.** Genetic relationships within Population1 (A) and Population2 (B) surveyed using STRUCTURE based on the 300 least correlated SNPs.

**
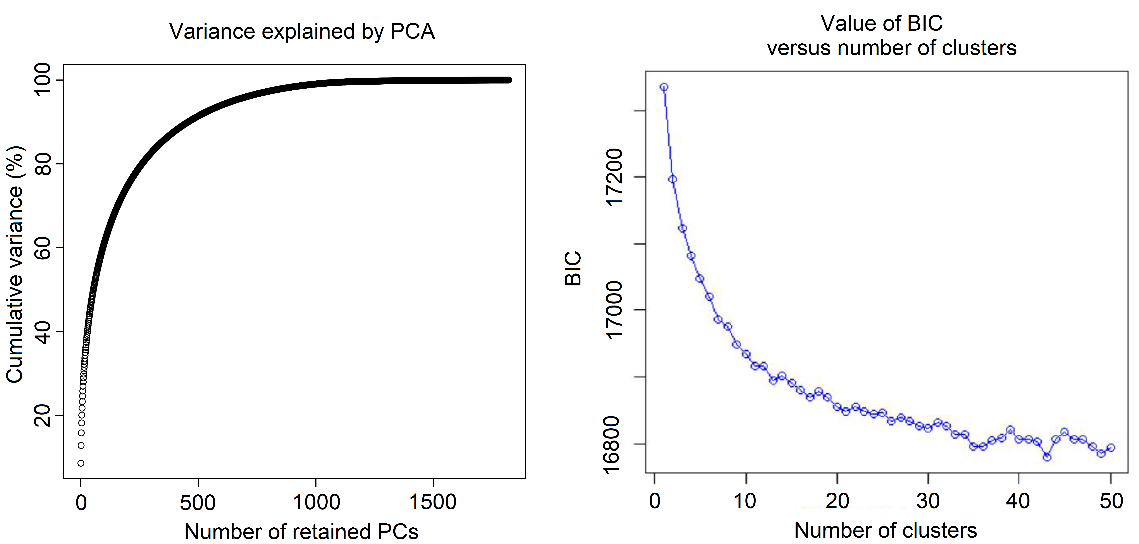
**

**Supplementary Fig 3.** Discriminant analysis of principal components (DAPC); (A) Variance explained by the principal components (extracted from genomic marker matrix); (B) Value of Bayesian Information Criterion (BIC) versus number of clusters.


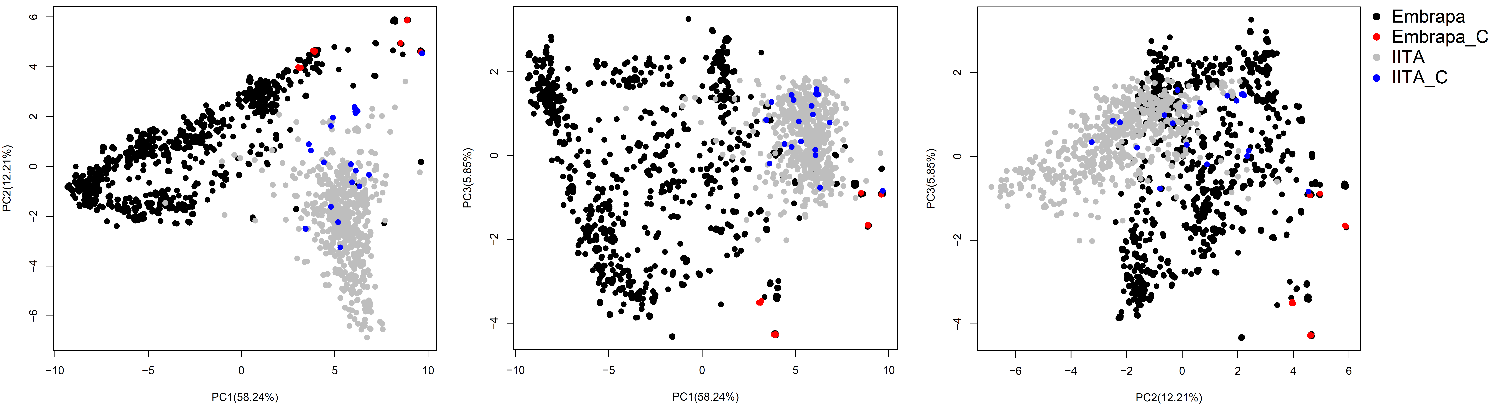


**Supplementary Fig 4.** Principal components analysis (PCA) based on the genomic kinship coefficients between clones: PCA derived from the genomic relationship matrix between all cassava clones (N=1,820; 14,924 SNPs) from Brazil (Embrapa) and Nigeria (IITA), showing the first three principal components and the variance explained by each component in parenthesis on the corresponding axis (58.24, 12.21 and 5.85% for PC1, PC2 and PC3, respectively); In black representing Embrapa clone’s dispersion and in grey representing IITA’s; Highlighting the coincident clones between own- and cross-country genomic predictions, in red representing Embrapa coincident clone’s dispersion and in blue representing IITA’s.
